# P-686. Risk of COPD Exacerbations, Asthma Exacerbations, and Hospitalizations for Heart Failure after RSV Hospitalization Among US Adults Aged at Least 50 Years

**DOI:** 10.1093/ofid/ofae631.882

**Published:** 2025-01-29

**Authors:** David Singer, Yan Wang, Aozhou Wu, Elizabeth M La, Susan Gerber, Lydia Lee, Hongjiao Liu, Keith A Betts

**Affiliations:** GSK, Philadelphia, Pennsylvania; Analysis Group, Los Angeles, California; Analysis Group, Los Angeles, California; GSK, Philadelphia, Pennsylvania; GSK, Philadelphia, Pennsylvania; GSK, Philadelphia, Pennsylvania; Analysis Group, Los Angeles, California; Analysis Group, Los Angeles, California

## Abstract

**Background:**

Older adults and adults with comorbidities are at increased risk of severe respiratory syncytial virus (RSV) disease. Limited data are available on the long-term impact of RSV on comorbid diseases. This study evaluated the association between RSV hospitalization and the risk of future acute disease worsening in adults aged ≥ 50 years.

Figure 1

**Figure:**
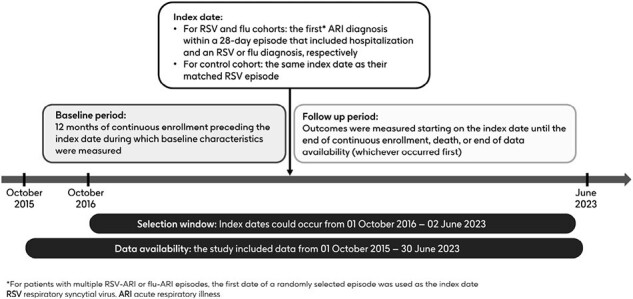

**Methods:**

This retrospective cohort study analyzed Optum’s de-identified Clinformatics Data Mart Database (CDM) healthcare claims from October 2015 to June 2023. Adults aged ≥ 50 years with ≥ 12 months of continuous enrollment were assigned to cohorts based on RSV hospitalization (RSV cohort), influenza hospitalization (flu cohort), or not having a recent acute respiratory illness (ARI) (controls). Index date was the start of an ARI episode that included hospitalization and an RSV or influenza diagnosis for the RSV and flu cohorts, respectively. Controls were matched 5:1 with the RSV cohort. Outcomes included acute exacerbations of (AE) chronic obstructive pulmonary disease (COPD), AE asthma, and hospitalizations for heart failure (HHF), were measured during the follow-up period (Fig. 1), and analyzed as recurrent events. AE COPD, AE asthma, or HHF risks were compared between the RSV vs influenza and RSV vs control cohorts using the Andersen-Gill formulation of Cox proportional hazards models in the overall population and subgroups.

**Figure:**
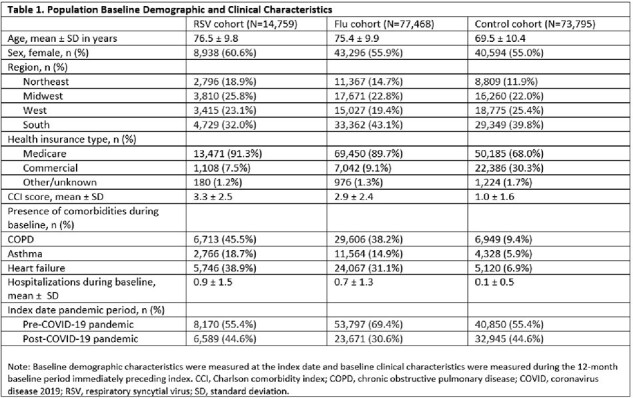

**Results:**

The study included 14,759, 77,468, and 73,795 adults in the RSV, flu, and control cohorts, respectively (Table 1). Mean cumulative event counts were highest in the RSV cohort (Table 2). In adjusted analyses, adults with an RSV hospitalization had a higher risk of AE COPD (for those with COPD: hazard ratio [HR] = 1.15 vs flu cohort, p < 0.001; HR = 2.78 vs control cohort, p < 0.001), AE asthma (for those with asthma: HR = 1.53 vs flu cohort, p < 0.001; HR = 6.51 vs control cohort, p < 0.001), and HHF (for those with heart failure: HR = 1.08 vs flu cohort, p < 0.001; HR = 3.07 vs control cohort, p < 0.001) (Table 3).

**Figure:**
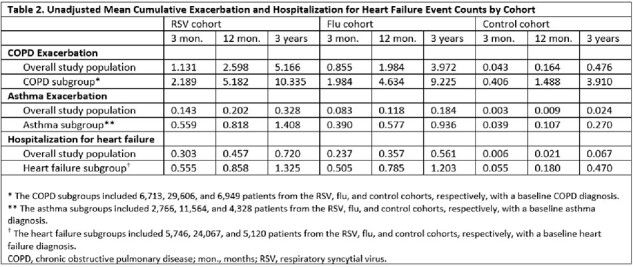

**Conclusion:**

Increased risk of AE COPD, AE asthma, and HHF was observed among adults with RSV hospitalization compared to influenza hospitalization and compared to adults without a recent ARI. These findings build on previous research demonstrating the importance of RSV prevention in older and comorbid adults.

FUNDING: GSK (VEO-000616)

**Figure:**
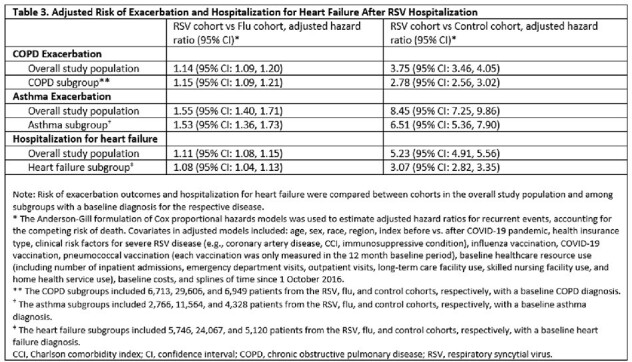

**Disclosures:**

**David Singer, PharmD, MS**, GSK: employee|GSK: Stocks/Bonds (Public Company) **Yan Wang, ScD**, Analysis Group: Employee **Aozhou Wu, PhD**, Analysis Group: Employee **Elizabeth M. La, PhD**, GSK: employee|GSK: Stocks/Bonds (Private Company) **Susan Gerber, MD**, GSK: Employee **Lydia Lee, PharmD, MS**, GSK: Fellowship Sponsorship **Hongjiao Liu, PhD**, Analysis Group: Employee **Keith A. Betts, PhD**, Analysis Group: Employee

